# The Long-Term Effects of High-Fat and High-Protein Diets on the Metabolic and Endocrine Activity of Adipocytes in Rats

**DOI:** 10.3390/biology10040339

**Published:** 2021-04-17

**Authors:** Ewa Pruszyńska-Oszmałek, Małgorzata Wojciechowska, Maciej Sassek, Hanna Krauss, Natalia Leciejewska, Dawid Szczepankiewicz, Piotr Ślósarz, Leszek Nogowski, Paweł A. Kołodziejski

**Affiliations:** 1 Department of Animal Physiology, Biochemistry and Biostructure, Poznan University of Life Sciences, 60-637 Poznan, Poland; maciej.sassek@up.poznan.pl (M.S.); natalia.leciejewska@up.poznan.pl (N.L.); dawid.szczepankiewicz@up.poznan.pl (D.S.); leszek.nogowski@up.poznan.pl (L.N.); 2 Department of Mother and Child Health, Poznan University of Medical Sciences, ul. Polna 33, 60-535 Poznań, Poland; malgorzata59@onet.eu; 3 Department of Medicine, The President Stanisław Wojciechowski State University of Applied Sciences in Kalisz, Nowy Świat 4, 62-800 Kalisz, Poland; hjk12@poczta.fm; 4 Department of Animal Breeding and Product Quality Assessment, Poznan University of Life Sciences, Sloneczna 1, 62-002 Zlotniki, Poland; piotr.slosarz@up.poznan.pl

**Keywords:** high-fat diet, high-protein diet, isolated adipocytes, adipokines, glucose uptake, lipolysis, lipogenesis

## Abstract

**Simple Summary:**

The increasing prevalence of worldwide obesity and growing awareness of its negative consequences are forcing scientists to take a new view of nutrition and search for new diets. Therefore, to find some new relationships between diet and metabolism, we analyzed the effects of the long-term (60 and 120 days) use of a high-protein diet (HPD) and of a high-fat diet (HFD) on the metabolic and endocrine functions of fat tissue and on biochemical indices in rat blood in the present study. This research helped us to understand the roles of diet in the metabolic and endocrine functioning of adipocytes. Our study indicated that an HFD has a negative effect on fat tissue function, whereas the HPD showed positive results, such as increased insulin sensitivity and improved glucose and lipid metabolism in isolated adipocytes in vitro.

**Abstract:**

The increasing prevalence of overweight and obesity and the rising awareness of their negative consequences are forcing researchers to take a new view of nutrition and its consequences for the metabolism of whole organisms as well as the metabolism of their individual systems and cells. Despite studies on nutrition having been carried out for a few decades, not many of them have focused on the impacts of these diets on changes in the metabolism and endocrine functions of isolated adipocytes. Therefore, we decided to investigate the effects of the long-term use (60 and 120 days) of a high-fat diet (HFD) and of a high-protein diet (HPD) on basic metabolic processes in fat cells—lipogenesis, lipolysis, and glucose uptake—and endocrine function, which was determined according to the secretion of adipokines into the incubation medium. Our results proved that the HPD diet improved insulin sensitivity, increased the intracellular uptake of glucose (*p* < 0.01) and its incorporation into lipids (*p* < 0.01) and modulated the endocrine function of these cells (decreasing leptin secretion; *p* < 0.01). The levels of biochemical parameters in the serum blood also changed in the HPD-fed rats. The effects of the HFD were inverse, as expected. We observed a decrease in adiponectin secretion and a diminished rate of lipogenesis (*p* < 0.01). Simultaneously, the secretion of leptin and resistin (*p* < 0.01) from isolated adipocytes increased. In conclusion, we noted that the long-term use of HPD and HFD diets modulates the metabolism and endocrine functions of isolated rat adipocytes. We summarize that an HFD had a negative effect on fat tissue functioning, whereas an HPD had positive results, such as increased insulin sensitivity and an improved metabolism of glucose and lipids in fat tissue. Moreover, we noticed that negative metabolic changes are reflected more rapidly in isolated cells than in the metabolism of the whole organism.

## 1. Introduction

The increasing prevalence of overweight and obesity and rising awareness of their negative consequences are forcing researchers to take a new view of nutrition and search for new diets. Recently, the impact of diet on metabolism and intake and storage of energy has been widely investigated. This is due to the increasing diagnosis of obesity among people and pets and the expanding trends of healthy lifestyles. If energy is supplied in excess, some of it may or must be stored as triglycerides in adipocytes. However, the adipose tissue not only works as a depot of fat and energy, but also produces and secretes adipokines, its own biologically active substances. They influence both appetite and energy homeostasis and regulate some aspects of the metabolism of carbohydrates and lipids. It is known that there is a relationship between the diet used and the metabolism of peripheral tissues, such as adipose tissue, liver, and skeletal muscle. So far, it has been shown that the long-term intake of a high-protein diet can increase liver triacylglycerol deposition pathways, which promote liver damage. Differently, a high-fat diet induces brown adipocyte gene expression in white adipose tissue [1,2]. Despite the fact that studies on nutrition have been carried out for a few decades [3,4], important issues such as the mechanisms responsible for the origins and development of obesity, metabolic syndrome, and insulin resistance are still not precisely clarified. In addition, the effects of the varied qualitative and quantitative compositions of nutrients in the diet on the metabolism of cells and tissues remain to be fully explored. To search for some new relationships between diet and metabolism, and to understand in detail the already known ones, we analyzed the effects of the long-term use of a high-protein (HPD) and of a high-fat diet (HFD) on the metabolic and endocrine functions of fat tissue and on the biochemical indices in rat blood in the present study. Lately, there has been growing interest in the roles of adipokines. Some of them, such as resistin, adiponectin, and leptin, are involved in the regulation of food intake and influence the biochemical parameters of the blood. In addition, together with insulin and glucagon, they play crucial roles in the regulation of appetite and metabolism and may affect the insulin sensitivity of tissues [5,6]. So far, it has been shown that resistin is able to influence the regulation of insulin secretion both during fasting and during feeding [7]; that a high-protein diet can modulate the expression and secretion of adiponectin [8]; and that a long-term HFD diet can lead to the phenomenon of leptin resistance [9].

This paper demonstrates some aspects of the metabolic and hormonal activity of adipocytes isolated from the white adipose tissue (WAT) of rats fed a standard diet (SD), an HPD, and an HFD for 60 and 120 days. It also describes the glucose uptake, lipogenesis, lipolysis, and secretion of adipokines (leptin, adiponectin, and resistin), as well as the hormonal and biochemical status of the animals fed different diets.

## 2. Materials and Methods

### 2.1. Materials

Unless otherwise stated, all the reagents used in this study were purchased from Sigma-Aldrich (Darmstadt, Germany). The reagents used in the experiments involving isotopes, carried out for analyzing the functions such as glucose uptake and lipogenesis, were purchased from Perkin Elmer (Norwalk, CT, USA).

### 2.2. Animals and Ethics

Male Wistar rats were used in this study. The animals were kept under standard conditions (12 h/12 h dark/light cycle, temperature 21 °C) before the experiments. All the experiments involving animals were approved by the local Ethical Commission for Animal Care and Use in Poznan, Poland (approval number 7/2009). After allowing them to adapt to the environment for 10 days, rats with an initial weight of 225.2 ± 4.33 g (age approximately 6 weeks) were divided into three groups and fed with different diets for 60 and 120 days (16 animals per diet: 8 animals per diet were sacrificed after 60 days and 8 after 120 days). The details about the diets are provided in a previous study [10]. In brief: Standard diet (2.75 kcal/g): crude protein 175 g; crude fat 2.8 g; crude fiber 70.0 g; starch 330.0 g; ash 57.0 g; calcium 9.5 g; total phosphorus 6.5 g; magnesium 3.0 g; potassium 7.5 g; sodium 1.9 g; sulfur 1.9 g; iron 144.0 mg; manganese mg 50.0; zinc 50.0 mg; copper 11.0 mg; iodine 0.2 mg; selenium 0.4 mg; vitamin A 12,000.0 IU; vitamin D3 800.0 IU; vitamin E 78.0 mg; vitamin K3 2.4 mg; vitamin B1 8.0 mg; vitamin B2 7.0 mg; vitamin B6 11.0 mg; vitamin B12 42.0 mcg; pantothenic acid 25.0 mg; folic acid 2.0 mg; biotin 0.3 mg; nicotinic acid 94.0 mg; choline 1900.0 mg; lysine 9.0 g; methionine + cyst 6.3 g; tryptophan 2.0 g; threonine 6.0 g; isoleucine 6.0 g; leucine 12.0 g; valine 8.0 g; histidine 4.0 g; arginine 10.0 g; phenylalanine 7.0 g; tyrosine 5.5 g; betaine 17.0 g. HFD diet (4.00 kcal/g)*:* standard diet + 20% fat (lard). HPD diet (3.53 kcal/g): standard diet + 20% soy protein. Animals were sacrificed by decapitation using a guillotine for small laboratory animals after 2 h fasting.

### 2.3. Glucose Tolerance Test (ipGTT)

Glucose tolerance test was performed four days before decapitation. After 6 h fasting, glucose (2 g/kg body weight) was injected intraperitoneally (ip) to animals. Glucose concentration was measured in blood drawn from tails 5 min before glucose injection, and at the indicated time points (5, 15, 30, 45, 60, 90 min). Glucose level was measured using glucometer AccuCheck Active (Roche Diagnostics GmbH, Mannheim, Germany). The trapezoidal rule was used to determine the area under the curve (AUC).

### 2.4. Adipocyte Isolation

Fat cells were isolated from the adipose tissue of rats using a procedure previously described by Rodbell [11] with some modifications. In brief, the excised peritoneal adipose tissue was washed with 0.9% NaCl at 37 °C, cut with scissors into small pieces, and digested in the Krebs–Ringer buffer (KRB) containing 3% bovine serum albumin, 10 mM HEPES, and 5 mM glucose with type-I collagenase (Sigma-Aldrich, Darmstadt, Germany) at 37 °C in a shaking water bath for 45 min. The solution obtained was filtered through a sterile nylon mesh (200 μm pore size). The isolated cells were washed three times using KRB without collagenase and then counted using a microscope and a Bürker-Türk’s counting chamber. The adipocytes were then used for the analysis of lipolysis, lipogenesis, and glucose uptake.

### 2.5. Lipolysis

Lipolysis was determined by quantifying the amounts of glycerol and nonesterified fatty acid (NEFA) released into the incubation medium. The isolated adipocytes were incubated for 120 min. Then, the incubation medium was collected for analysis, and the concentration of glycerol and NEFA in the medium was determined using free glycerol reagent (Sigma-Aldrich) and NEFA kit (Wako Diagnostics, Mountain View, CA, USA).

### 2.6. Lipogenesis and Glucose Uptake

Lipogenesis and glucose uptake analyses were performed as we previously described [12,13]. Briefly, lipogenesis was studied by determining the incorporation of [U-14c] glucose into the lipids. Isolated adipocytes were incubated with KRB buffer with the addition of 0.5 µCi [U-^14^C] glucose in the absence (basal lipogenesis) or presence (insulin stimulated lipogenesis) of insulin [10 nM] for 120 min. The reaction was stopped using an ice cold Dole mixture [14]. Lipids fraction was separated using Dole method. Separation of the lipid phase was performed by adding H_2_O and heptane. Then, the lipid fraction was transferred into a scintillation liquid for counting of incorporated radioactivity using a β-counter (TRI-CARB Liquid Scintillation Counter, Perkin Elmer; Norwalk, CT, USA).

Glucose uptake was determined using deoxy-D-glucose, 2-[14C(U)]. In brief, adipocytes isolated from rats were incubated in a water bath with (stimulated) or without (basal) insulin. Subsequently, 0.5 µCi [U-^14^C] glucose was added to adipocytes for 5 min. To determine the concentration, ice cold KRB buffer supplemented with 1.5 mM of cytochalasin B was added. 

### 2.7. Determination of Adiponectin, Leptin, and Resistin in Serum and Incubation Medium

The concentrations of adiponectin, resistin, and leptin were measured using commercially available ELISA and RIA kits on the incubation medium samples collected for the determination of lipolysis. The following were used to determine the concentrations of peptides: the Multi-Species Leptin RIA Kit (catalog number XL-85K, Merck Millipore, Burlington, MA, USA), Rat Resistin ELISA Kit (catalog number EIAR-RES-1, RayBiotech Life, Peachtree Corners, GA, USA), Rat Adiponectin ELISA Kit (catalog number ELR-Adiponectin-1, RayBiotech Life, Peachtree Corners, GA, USA), and Rat Visfatin ELISA Kit (catalog number EIAR-VIS-1, RayBiotech Life, Peachtree Corners, GA, USA). The serum used for analysis was taken postmortem. Animals were sacrificed using a guillotine for small laboratory rodents. The blood collected from animals was left for 20 min to clot. Then, the samples were centrifuged for 15 min at 3500× *g* at 4 °C. After centrifugation, the serum was collected into new tubes and stored at 80 °C for further analysis.

### 2.8. Metabolic Profile in Blood Serum

The metabolic profile in serum blood was determined using commercially available colorimetric tests according to manufacturers’ instructions. The list of kits together with their catalog numbers and the producers are presented here: glucose (catalog number G7519), triglycerides (catalog number T7531), cholesterol (catalog number C7510) (Pointe Scientific, Canton, MI, USA), NEFA (catalog number HR Series NEFA-HR, Wako diagnostics, USA). Insulin and glucagon levels in sera were measured using RIA kits. (Rat insulin RIA kit, catalog number RI-13K; Glucagon RIA kit, catalog number GL-32K, Merck Millipore, USA).

### 2.9. Statistical Analysis

Data are presented as means ± SEM with individual values. Statistical analyses were performed using GraphPad Prism 6 Software and one-way ANOVA followed by the Tukey post-hoc test or the Kruskal–Wallis, and Dunn tests were used to analyze the nonparametric results. Statistical analyses were carried out only between different diets within one time point. Statistical significance was accepted if * *p* < 0.05, ** *p* < 0.01. 

## 3. Results

### 3.1. Body Weight and General Metabolic Profile

**Figure 1:**
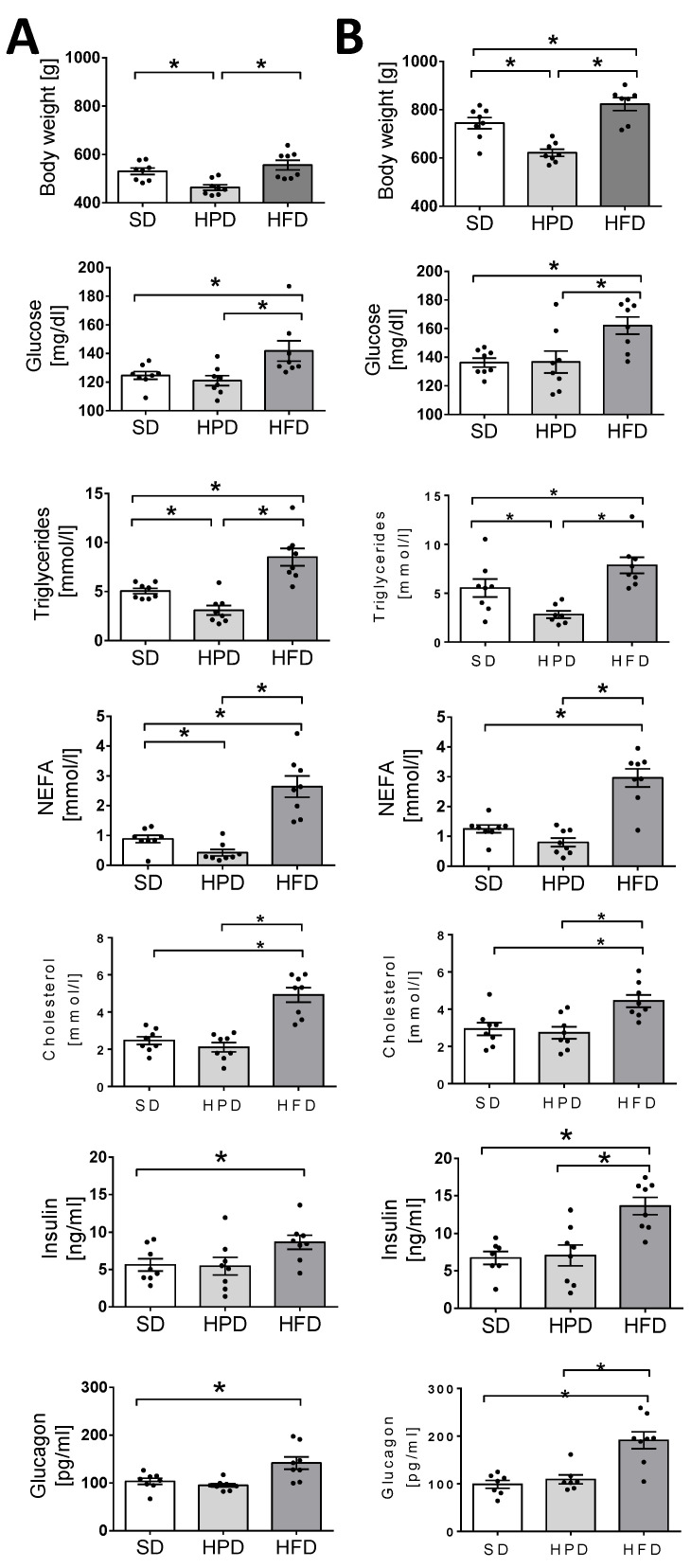
The effects of high-fat diet (HFD) and high-protein diet (HPD) treatment on body weight, glucose, triglycerides, NEFA, cholesterol, insulin, and glucagon concentrations in serum blood after 60 days (panel **A**) and 120 days of treatment (panel **B**). Results are means ± SEM for n = 8; significant differences between means for control and experimental groups are marked for *p* < 0.05 (*).

To characterize the effects of an HFD and HPD on the metabolic profile, in comparison to an SD, we measured the body mass and the concentrations of glucose, triglycerides, NEFA, and cholesterol in the sera of the rats treated with these diets (Figure 1).

As expected, the diets applied ad libitum differently influenced the body weights of the animals. The rats treated for 60 and 120 days with the HPD had the lowest weight among the groups. The body mass of the animals kept on an HFD did not differ from that of the rats on the SD up to the 60th day. However, on the 120th day, the HFD-treated rats were significantly heavier than the SD-treated rats (Figure 1). Simultaneously, disturbances were observed in the glucose metabolism in the HFD group from day 60 and the level of glucose in the serum was increased significantly at both the investigated time points. By contrast, no negative effects were observed in the rats fed the HPD and the level of glucose in the serum was the same as in the SD group. Similarly, the investigated diets exerted different and in fact contrasting effects on the lipid parameters. The serum concentrations of triglycerides and NEFA were considerably increased in the HFD group and decreased in the HPD group. In addition, a high increase in the level of cholesterol was observed in the HFD group, whereas the HPD was not associated with any significant change in this parameter.

### 3.2. Glucose Utilization Rate

**Figure 2:**
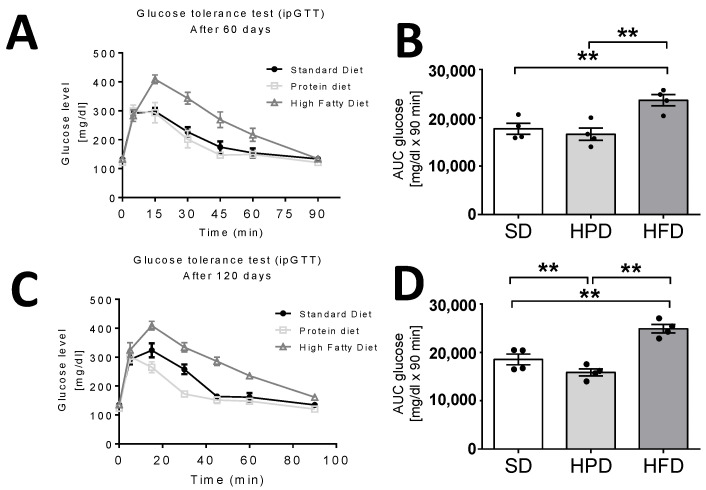
The effects of three different diets on glucose utilization rate (*ip*GTT) after 60 (**A**,**B**) and 120 days (**C**,**D**). (**B**,**D**) show mean areas under glucose curves. Results are means ± SEM for n = 4; significant differences are marked for *p* < 0.01 (**).

Glucose tolerance tests (Figure 2) showed the negative influence of an HFD on glucose utilization. During the tests, the glucose absorption rate from blood into the tissues was found to be lowest in the HFD group compared to the other groups (SD, HPD vs. HFD; *p* < 0.01). The mean area under the glucose curve was significantly elevated in the HFD group on days 60 and 120, indicating the impairment of the mechanisms regulating glucose homeostasis (*p* < 0.01). On the contrary, the rats kept for 120 days on the HPD utilized glucose statistically significantly better than the animals in the other groups, including those fed the SD (*p* < 0.01).

### 3.3. Impacst of the Diets on Hormone Concentrations in the Serum

**Figure 3:**
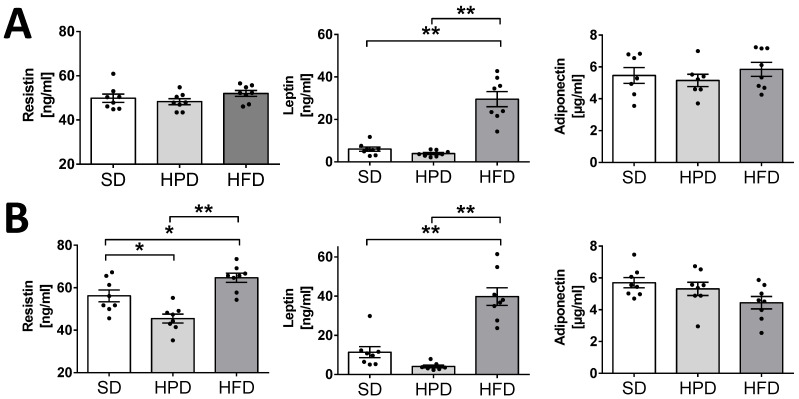
The effects of the standard (SD), HFD, and HPD on resistin, adiponectin, and leptin concentrations in blood serum after 60 (**A**) and 120 (**B**) days. Values presented are means and SEM for n = 8; significant differences between means for control and experimental groups are marked for *p* < 0.05 (*) and *p* < 0.01 (**).

The HFD applied ad libitum not only influenced the important biochemical parameters of the blood, but also caused significant endocrine changes. This diet caused significant elevations in the concentrations of insulin and glucagon on days 60 and 120 (Figure 1). On day 120, the level of insulin was increased by over 100% and glucagon by over 90% in the HFD group in comparison to the animals kept on the SD (*p* < 0.05). No statistically significant differences were noticed in the levels of these hormones between the HPD and SD-treated rats. In addition to these, significant changes were also observed in leptin content in the sera of the HFD-treated rats. On the 60th and 120th days, these animals showed several-fold higher levels of leptin than the rats kept on the SD and HPD (Figure 2; *p* < 0.01), and the changes were statistically significant. No differences were observed between the SD and HPD groups. On the other hand, the levels of resistin in the different groups were almost the same on day 60; however, on day 120, the HFD-treated rats showed a slightly, but statistically significantly, elevated concentration (Figure 2; *p* < 0.05). Moreover, the concentration of resistin in the HPD group after 120 days was lower compared to SD (*p* < 0.05) and HFD (*p* < 0.01). In contrast to the above-presented results, no significant changes were observed in the serum levels of adiponectin and visfatin (Supplementary Figure S1) among the groups, and the levels were almost consistent throughout the experiment (Figure 3).

### 3.4. Leptin, Resistin, and Adiponectin Secretion from Isolated Rat Adipocytes

**Figure 4:**
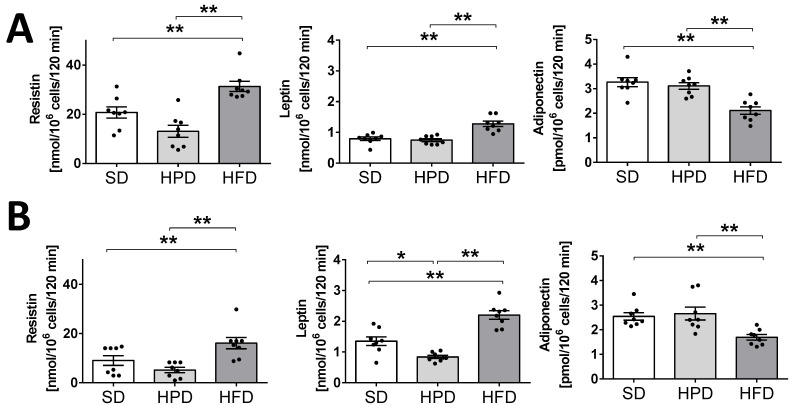
The effects of high-protein and high-fat diets on resistin, adiponectin, and leptin secretion from isolated rat adipocytes after 60 (**A**) and 120 days (**B**). Cells were incubated for 2 h in Krebs–Ringer buffer; hormones’ concentrations in the medium were measured using Elisa kits. Results are means ± SEM for n = 8; significant differences between means for control and experimental groups are marked for *p* < 0.05 (*) and *p* < 0.01 (**).

We examined the effects of the HFD and HPD on the endocrine function of isolated adipocytes by determining the secretion of adipokines such as leptin, adiponectin, and resistin into the incubation medium (Figure 4). Adipocytes isolated from the HFD-treated rats were found to secrete significantly more leptin and resistin at both the time points (days 60 and 120; *p* < 0.01), but less adiponectin (*p* < 0.01) compared to the SD. Adipocytes obtained from the animals kept on the HPD for 60 and 120 days showed lower secretion of resistin, but the differences were not statistically important. However, the reduced secretion of leptin observed after 120 days was statistically significant (*p* < 0.05). The in vitro release of leptin and resistin by the isolated adipocytes was convergent with the elevation of these hormones observed in vivo in the HFD-treated rats and their diminution in the HPD-treated rats. Furthermore, these results corresponded well with the content of leptin in the peritesticular fat as well as in the subcutaneous fat tissue of the rats on the SD, HPD, and HFD. The amounts of adiponectin secreted to the medium by the adipocytes of SD and HPD groups were comparable; however, incubation medium obtained from HFD adipocytes showed lower concentrations of adiponectin after 60 and 120 days compared to SD and HPD groups (*p* < 0.01). Other analyses indicated a high content (*w*/*w*) of leptin in the peritesticular and subcutaneous WAT of the rats kept on the HFD for 120 days (Table S1). By contrast, the amount (*w*/*w*) of this hormone was observed to be low in the peritesticular WAT of the rats on HPD. On the 120th day, this result was statistically significant compared to the SD and HFD groups. The levels of resistin (*w*/*w*) in the subcutaneous fat tissue of the rats kept on the HPD and HFD never exceeded the values observed for the SD-treated rats. Interestingly, the 120-day-long feeding with HPD or HFD caused a significantly lower expression of resistin compared to the rats on SD. In peritesticular fat, a significantly lower content of this hormone was observed on only the 60th day in the HPD-treated rats. However, a statistically significant increase in the level of this hormone (*w*/*w*) was observed in the pancreases of these animals on both the 60th and 120th days (Table S1).

### 3.5. Impact of the Diets on Lipolysis, Lipogenesis, and Glucose Uptake by Isolated Rat Adipocytes

**Figure 5:**
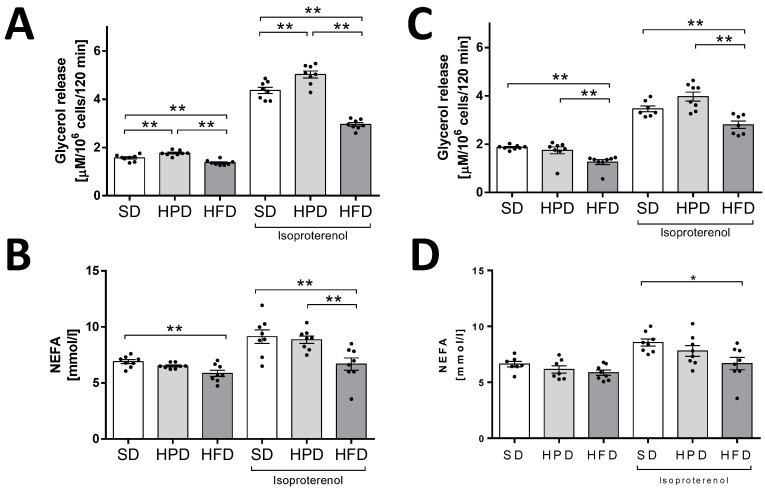
The effects of the standard diet, HFD, and HPD on basal and isoproterenol stimulated lipolysis in isolated rat adipocytes after 60 (**A**,**B**) and 120 (**C**,**D**) days. Intensity of lipolysis was measured as glycerol and NEFA release from cells into incubation medium. Results are means ± SEM for n = 8; significant differences between means for control and experimental groups are marked for *p* < 0.05 (*) and *p* < 0.01 (**).

To characterize the sensitivity and metabolic activity of adipocytes, processes such as glucose uptake, lipolysis, and lipogenesis were analyzed. The intensity of basal and isoproterenol stimulated lipolysis was determined. The indicators of lipolysis were glycerol and NEFA released from adipocytes into the incubation medium (Figure 5). Considering the amount of glycerol released, both basal and stimulated lipolysis were found to be decreased in the HFD group. Convergent results confirming the slower rate of lipolysis in the HFD group were observed when the amount of NEFA released in the medium was estimated to be the lowest under both the experimental conditions (basal and stimulated). These changes, except the rate of basal lipolysis on the 120th day, were statistically significant in comparison to the SD group. All these results indicated that the HFD decreased the rates of basal and stimulated lipolysis. At the same time, this diet favored statistically significant basal and insulin-stimulated transport of labeled 2-deoxyglucose (2-DG) and lipogenesis, which was measured as radioactivity incorporated into lipids from the labeled glucose.

**Figure 6:**
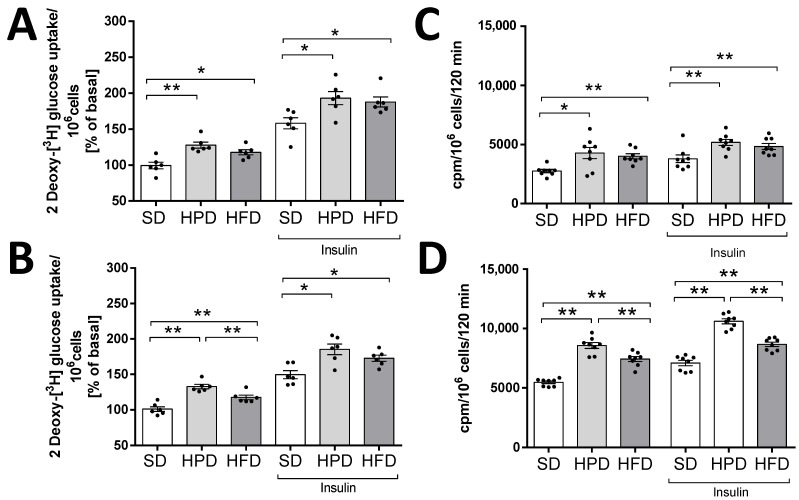
Effects of standard (SD), high-protein (HPD), and high-fat (HFD) diets on glucose uptake and lipogenesis in isolated adipocytes after 60 (**A**,**C**) and 120 days (**B**,**D**). Results are means ± SEM for n = 8 (lipogenesis experiments) and n = 6 (glucose uptake); significant differences between means for control and experimental groups are marked for *p* < 0.05 (*) and *p* < 0.01 (**).

In comparison to the HFD, the HPD did not univocally affect lipolysis (Figure 5). A faint stimulation of lipolysis was observed only when the glycerol content was estimated in the incubation medium after 60 days of HPD treatment. Simultaneously, adipocytes isolated from the rats in the HPD group were found to be strongly responsive to glucose (Figure 6). The uptake of 2-DG by adipocytes and glucose conversion into lipids were the highest in the HPD-treated animals compared to the other groups. 

Increases of the intensity of basal and insulin stimulated lipogenesis were observed in the HFD and HPD groups compared to the control group after both 60 and 120 days of using these diets. In addition, it was shown that the most lipids were synthesized in adipocytes isolated from adipose tissue of the HPD-fed rats (after 120 days of the experiment, this group differed significantly from SD and HFD groups) (Figure 6C,D).

Similarly, there were increases in the rate of intracellular glucose uptake in the HFD and HPD groups compared to the control group (SD-fed rats) for both the basal and insulin stimulated processes. Furthermore, by analyzing the effect of using the HPD diet, we found that the rate of basal glucose transport to fat cells was significantly higher than that in the HFD group after 120 days of the experiment (Figure 6A,B).

## 4. Discussion

The main purpose of this study was to compare the long-term effects of a SD, HPD, and HFD on the metabolic and endocrine activity of white fat tissue, as important components of the whole-body processes initialized by nutrients. The paper presents the results of the study conducted on isolated adipocytes and collected tissues, which, together with the in vivo measurements, enabled a broad analysis of the effects of the tested diets. The influences of various diets were investigated in many previous studies, often with the aim of transposing the results from animals to humans. The physiology of animals may not correspond ideally to that of the humans, but animals are considered the simplest and most useful physiological models for testing different diets and disorders [15]. However, not all the effects of high-fat or high-protein feeding are known at the cellular, molecular, biochemical, and endocrine levels. The results presented by us show the different effects of diets (HFD and HPD) on intracellular glucose uptake and its incorporation into lipids; the endocrine profile and biochemical parameters in the serum; and the activity of adipocytes, including some aspects of their metabolism (lipolysis, lipogenesis, and glucose incorporation) and the secretion of adipokines. It is often supposed that metabolic and endocrine (dys)functions of the adipose tissue play roles in obesity, metabolic syndrome, and diabetes. Therefore, an investigation of fat tissue aiming at the characterization of its intracellular turnover and secretory activity, as well as its relationship with the metabolic processes of the whole body, may widen the knowledge on the physiological significance of this tissue and its associations with the important functions of the body and some disorders. At both the chosen time points (days 60 and 120), the mean body weight of rats kept (for 60 and 120 days) on the HPD was found to be considerably below that of the animals fed the SD or the HFD (Figure 1). These results are consistent with the effects previously shown in a study by Lacroix et al., which compared the effects of the long-term administration of a SD and an HPD (3 and 6 months) in rats [11]. The decrease in body weight in animals receiving the HPD (50% protein) compared to animals fed with the SD (14% protein) was due to a decreased WAT content, possibly due to a lower feed intake. Additionally, in animals from the HPD group, a lower concentration of TG in the blood serum was observed, which is consistent with our results (Figure 1). Through some in vitro investigations, we tried to shed light on the general metabolism, including the specific metabolism of the adipose tissue. First, we observed better glucose tolerance and hence better glucose utilization in the animals on the HPD (Figure 2). The extent to which this may affect the turnover of lipids in adipocytes is unknown. Paradoxically, except for the increase in the release of glycerol into the incubation medium on day 60 of the experiment, we did not observe an influence of the HPD on the intensity of lipolysis (Figure 3). In addition, at the cellular level, increased uptake of 2-DG into adipocytes and increased incorporation into lipids were observed in the conditions of both basal and insulin-stimulated lipolysis (Figure 5). These results are astonishing considering the lower body mass of rats kept on the HPD and the previous observations of decreased fat content [16]. The possible explanation may be that during long-term feeding with HPD, the adipocytes isolated from the HPD-treated rats were metabolically very active, “hungry,” and more sensitive to convert every dose of glucose to fat. However, this euphemism does not explain the molecular basis of this state, and it is logical that feeding with an excess of proteins strongly unbalances the regular sources of energy in the fodder, and forces the organism to utilize amino acids from proteins as an important or even primary fuel. Simultaneously, this situation probably forces the organism to save lipids, which, under the natural in vivo conditions, are slowly converted to glycerol and free fatty acids in the adipose tissue and released into the blood. This may be a reason for the diminished level of NEFA observed. Furthermore, the adipocytes of the HPD-treated rats were able to effectively utilize the glucose to synthesize lipids, and this phenomenon was only observed in the in vitro condition.

Our results demonstrate efficient glucose utilization and tolerance in HPD-fed rats (Figure 2). Similarly, Lacroix et al. observed lower insulin levels and better oral glucose tolerance in comparison to rats fed a “normal” diet, which is a sign of proper carbohydrate metabolism. They suggested also that the long-term consumption of an HPD may also prevent metabolic syndrome. Earlier, Pichon et al. found lower weight gain and less body weight for HPD-fed rats, as well as a lower body fat index, a smaller amount of adipose tissue, and a smaller average diameter for the adipocytes [16].

The contrasting effects of the HFD and HPD, among other things, on body mass, are clearly shown in the present paper. However, the onset of this effect was found to be delayed. Up to the 60th day, no additional increase in the body mass of rats on the HFD was observed in comparison to the rats fed the SD (but also ad libitum). The acceleration of body weight gain was noted between the 60th and the 120th days, and the animals fed the HFD were significantly heavier. The final effect of the HFD on body mass observed in our experiments was in line with the results of the investigations reported by Kubota et al. on mice [3]. They found that the animals kept on the HFD were heavier and stored more WAT in comparison to those kept on a high-carbohydrate diet due to the bigger size of adipocytes. In addition, Kim et al. indicated the same trend, concluding that HFD consumption increases body weight and augments the amount of fat [17]. It has been also reported/well documented that the HFD is a contributing factor in the development of obesity in rats. Higher levels of dietary fat increase the susceptibility to lipid accretion in fat tissue, which results in more and larger adipocytes [18,19,20,21,22].

Previous studies showed that the type of diet can affect, inter alia, the hypertrophy of fat cells mediated by PPARγ, blood leptin, and the activity of liver fatty acid synthase, but not lipoprotein lipase in WAT [3,23]. However, the rate of TG accumulation is also regulated by lipolysis and lipogenesis. We showed for the first time the direct effects of high-fat and high-protein diets on these metabolic processes in isolated rat adipocytes. Here, we have found that the HFD and HPD modulated the intensity of lipolysis after 60 and 120 days (Figure 3). The HFD diminished the release of glycerol from adipocytes and fatty acids on day 60. Reduction in the activity of the adipose triglyceride lipase (AGLT) (enzyme which catalases the TG decomposition) may be the reason for the decrease in the intensity of lipolysis in adipocytes isolated from HFD rats. As reported by Oliver et al., the diet-induced obesity decreased ATGL expression (mRNA and protein levels) in different adipose depots [24]. This was overlapped by the increased transport of glucose into the cells and its enhanced incorporation into the lipids (Figure 4). Simultaneously, adipocytes from HFD-treated rats showed less sensitivity to lipolytic stimuli (isoproterenol); we can even say that they were almost resistant (Figure 4). Thus, some mechanisms leading to the increase in fat reserves are obvious. Additionally, our investigations indicated that when the HFD or HPD was used in the long term, the adipocytes responded to insulin with an increased uptake of glucose and by elevated lipogenesis. However, adipocytes isolated from HPD-fed animals showed a higher rate of lipolysis compared to cells isolated from HFD-fed rats. These observations are significant when explaining the physiological changes that occur during daily activities. Going further, these may be fundamental for understanding the mechanisms of weight cycling or yo-yo effects observed after changing diet. Moreover, we stated that the consumption of an HFD is accompanied by an increase in the concentration of the proanabolic and anticatabolic hormone insulin in the blood (Figure 1). The concentration of this hormone observed in the rats on HFD was about twice as much as that in the SD-fed animals. Such multilateral relations lead to increased body weight and constitute the source of metabolic disturbances (Figure 1), including the significant elevation of glucose, triglycerides, NEFA, and cholesterol in the blood. At the same time, in the animals on the HFD, the secretion of glucagon was not suppressed completely by the action of insulin, and a high level of glucagon observed in the blood may have propelled the secondary changes expressed as enormously elevated concentrations of glucose and lipids in the blood. From the 60th day, an increase in tolerance to glucose was visible. The results indicate how dangerous the overconsumption of lipids is, and how easy, simple, and fast the induction of obesity, metabolic syndrome, and diabetes is (Figure 5 and Figure 6). Both the secretion of leptin by adipocytes and the blood concentration of this hormone are extremely elevated by a fat-rich diet. Those things indicate that the organism should reduce consumption. Moreover, we found a higher content of thishormone in the peritesticular and subcutaneous adipose tissue of HFD-fed animals compared to SD and HPD groups (Table S1). It is known that a high level of blood leptin in obesity leads to the development of resistance to this hormone in the brain, and therefore suppressing the feeding cannot reduce its blood concentration. In the literature, we can find enough data demonstrating the elevated level of leptin associated with ad libitum overconsumption followed by leptin resistance [25,26,27]. This is proved in the case of fat livestock and laboratory pets [28]. An unfavorable effect accompanying the elevation of body mass and increased secretion of leptin in the HFD-fed rats is the heightened level of resistin in the blood (Figure 5) which is most probably the result of its increased secretion from adipocytes (Figure 6). An increased level of both the abovementioned hormones together with insulin will lead to deleterious effects. Most often, resistin is regarded as a proinflammatory and prodiabetic agent [6,29], and the elevation of leptin is associated with the rise of resistin [30]. This is observed in cases of obesity, insulin resistance, metabolic syndrome, and diabetes [31]. In addition, we observed that adipocytes of the HFD-fed rats produced a smaller amount of adiponectin, another adipokine whose level is negatively correlated with a tendency toward diabetes [29], while its reduced secretion from cells led to negative consequences. Thus, as we have also shown, the ad libitum intake of HFD may lead, directly, to the development of obesity, insulin resistance, and metabolic syndrome, which are complex and multilateral phenomena. One of the potentially significant observations that is difficult to interpret is the result showing an increase in resistin content in the pancreases of animals fed with an HPD (Table S1). In our previous work, we showed that resistin is secreted by alpha cells [29,30]. However, in the present study it is difficult to predict the consequences for the pancreas of a higher concentration of resistin in animals treated with HPD. This action of resistin may be interpreted in two ways: (i) positive—considering its relatively high level in the pancreases of the HPD-treated rats, it may limit insulin secretion and save this organ via paracrine intrapancreatic and intraislet mechanisms adapting the pancreatic endocrine action to a diet with a relatively low percentage of sugars; (ii) negative—despite the positive effect on the limitation of insulin secretion, it might have increased the proinflammatory action within the pancreas [32,33]. Summarizing all the metabolic and endocrine results, we may say that the ad libitum HFD (including carbohydrates) is certainly harmful to rat health. Besides a high increase in body mass, it caused large increases in the levels of glucose, triglycerides, NEFA, cholesterol, insulin, glucagon, and leptin, and a slight increase in the level of resistin in the blood. Feeding on this diet led to impairment of glucose tolerance in vivo. Furthermore, we observed decreased lipolysis intensity with simultaneous increases in the rates of glucose uptake and lipogenesis in isolated adipocytes in vitro. In addition, adipocytes secreted less adiponectin; however, we did not observe the lower level of this hormone in vivo in the blood.

On the contrary, the main action of ad libitum HPD is metabolically positive for rat health. Some results did not differ from those observed for standard ad libitum feeding—serum levels of glucose, cholesterol, insulin, and glucagon; some were better than those observed on a SD—diminished concentrations of triglycerides, NEFA, and leptin in the serum. In addition, the HPD-treated animals showed better glucose tolerance. A few in vitro effects caused by both the diets were very similar—increases of basal and insulin-stimulated glucose transport to adipocytes and incorporation into lipids; however, the reactivity of isolated cells was greater in the case of HPD. Surprisingly, against what would be expected, basal and isoproterenol-stimulated lipolysis in adipocytes did not increase evidently in the HPD group. 

Our study has several limitations. First of all, we did not investigate other health factors, such as hepatic and/or kidney function, after the use of the HFD and HPD, as well as the intracellular metabolic pathways that are associated with the observed changes. We are also aware that the rat model is not ideal for this type of research and the results obtained with such a model can only be partially and carefully transposed to humans. However, in this study, we focused on the metabolic changes observed in the hormonal profiles of rats, and the endocrine and metabolic activity of adipose tissue in vitro.

## 5. Conclusions

In conclusion, we found that the long-term use of the HFD or HPD modulates the functioning of adipose tissue and isolated adipocytes. However, we are aware of a few limitations of our study. First limitation of our study is that the diet which we used in the study was not balanced (fat or protein were added on the top on standard diet), which does not allow for drawing conclusions about individual nutrients, but for a general conclusion about diets as whole nutrition. On the other hand, this limitation can be considered as an advantage, especially in the context of relating the results to changes caused by unhealthy diet in human. We believe that such a set of experiments more closely simulates the situation of poor nutrition in which increasing the consumption of one of the components of the diet usually does not go hand in hand with additional balancing of the diet. Another limitation of our research is the lack of determining the specific metabolic pathways responsible for these changes, as well as the fact that it focuses only on adipose tissue, which does not allow us to look at other possible negative effects of the HPD diet, such as kidney damage caused by excess protein in the diet. In addition, the advantage of our study is the long-term use of both diets in the same experiment, which allows the effects of diets to be compared without taking into account other research factors, such as age or coloration of rats. However, despite the several limitations of these studies, we believe the results are significant in terms of both the use of different diets and their effect on metabolism and this is new knowledge that can be used as the basis for further research. Taken together, we demonstrated that the HFD had a negative effect on fat tissue functioning, whereas the HPD exhibited positive results such as an increase in insulin sensitivity and improved metabolism of glucose and lipid in the adipose tissue.

## Data Availability

The data presented in this study are available on reasonable request from the corresponding author.

## Supplementary Materials

The following are available online at https://www.mdpi.com/article/10.3390/biology10040339/s1, Figure S1: Effect of HPD and HFD on visfatin serum level after 60 (A) and 120 days (B) of diet treatment, Table S1: Content of adipokines in white adipose tissue (Pt-peritesticular; Sc-subcutaneous) and rat pancreas (Pc) on standard (SD), high protein (HPD), and high fat (HFD) diets on 60 and 120 days.

## References

[1] Díaz-Rúa R., Keijer J., Palou A., van Schothorst E.M., Oliver P. (2017). Long-term intake of a high-protein diet increases liver triacylglycerol deposition pathways and hepatic signs of injury in rats. J. Nutr. Biochem..

[2] García-Ruiz E., Reynés B., Díaz-Rúa R., Ceresi E., Oliver P., Palou A. (2015). The intake of high-fat diets induces the acquisition of brown adipocyte gene expression features in white adipose tissue. Int. J. Obes..

[3] Kubota N., Terauchi Y., Miki H., Tamemoto H., Yamauchi T., Komeda K., Satoh S., Nakano R., Ishii C., Sugiyama T. (1999). PPARγ mediates high-fat diet-induced adipocyte hypertrophy and insulin resistance. Mol. Cell.

[4] Ntambi J.M., Kim Y. (2000). Adipocyte Differentiation and Gene Expression. J. Nutr..

[5] Smitka K., Marešová D. (2015). Adipose Tissue as an Endocrine Organ: An Update on Pro-inflammatory and Anti-inflammatory Microenvironment. Prague Med. Rep..

[6] Galic S., Oakhill J.S., Steinberg G.R. (2010). Adipose tissue as an endocrine organ. Mol. Cell. Endocrinol..

[7] Oliver P., Ribot J., Rodríguez A.M., Sánchez J., Picó C., Palou A. (2006). Resistin as a putative modulator of insulin action in the daily feeding/fasting rhythm. Pflugers Arch. Eur. J. Physiol..

[8] Nagasawa A., Fukui K., Funahashi T., Maeda N., Shimomura I., Kihara S., Waki M., Takamatsu K., Matsuzawa Y. (2002). Effects of soy protein diet on the expression of adipose genes and plasma adiponectin. Horm. Metab. Res..

[9] Sáinz N., Barrenetxe J., Moreno-Aliaga M.J., Martínez J.A. (2015). Leptin resistance and diet-induced obesity: Central and peripheral actions of leptin. Metabolism.

[10] Nowacka-Woszuk J., Pruszynska-Oszmalek E., Szydlowski M., Szczerbal I. (2017). Nutrition modulates Fto and Irx3 gene transcript levels, but does not alter their DNA methylation profiles in rat white adipose tissues. Gene.

[11] Rodbell M. (1964). Metabolism of isolated fat cells. 1. Effects of hormones on glucose metabolism and lipolysis. J. Biol. Chem..

[12] Pruszynska-Oszmalek E., Kolodziejski P.A., Kaczmarek P., Sassek M., Szczepankiewicz D., Mikula R., Nowak K.W. (2018). Orexin A but not orexin B regulates lipid metabolism and leptin secretion in isolated porcine adipocytes. Domest. Anim. Endocrinol..

[13] Pruszyńska-Oszmałek E., Kołodziejski P.A., Sassek M., Sliwowska J.H. (2017). Kisspeptin-10 inhibits proliferation and regulates lipolysis and lipogenesis processes in 3T3-L1 cells and isolated rat adipocytes. Endocrine.

[14] Dole V.P., Meinertz H. (1960). Microdetermination of Long-chain Fatty Acids in Plasma and Tissues. J. Biol. Chem..

[15] Engel H., Xiong L., Reichenberger M.A., Germann G., Roth C., Hirche C. (2019). Rodent models of diet-induced type 2 diabetes mellitus: A literature review and selection guide. Diabetes Metab. Syndr. Clin. Res. Rev..

[16] Lacroix M., Gaudichon C., Martin A., Morens C., Mathé V., Tomé D., Huneau J.F. (2004). A long-term high-protein diet markedly reduces adipose tissue without major side effects in Wistar male rats. Am. J. Physiol. Regul. Integr. Comp. Physiol..

[17] Kim J.Y., Nolte L.A., Hansen P.A., Han D.H., Ferguson K., Thompson P.A., Holloszy J.O. (2000). High-fat diet-induced muscle insulin resistance: Relationship to visceral fat mass. Am. J. Physiol. Regul. Integr. Comp. Physiol..

[18] Rothwell N.J., Stock M.J. (1984). The development of obesity in animals: The role of dietary factors. Clin. Endocrinol. Metab..

[19] Ghibaudi L., Cook J., Farley C., van Heek M., Hwa J.J. (2002). Fat intake affects adiposity, comorbidity factors, and energy metabolism of sprague-dawley rats. Obes. Res..

[20] Buettner R., Schölmerich J., Bollheimer L.C. (2007). High-fat diets: Modeling the metabolic disorders of human obesity in rodents. Obesity.

[21] Poret J.M., Souza-Smith F., Marcell S.J., Gaudet D.A., Tzeng T.H., Braymer H.D., Harrison-Bernard L.M., Primeaux S.D. (2018). High fat diet consumption differentially affects adipose tissue inflammation and adipocyte size in obesity-prone and obesity-resistant rats. Int. J. Obes..

[22] Bastías-Pérez M., Serra D., Herrero L. (2020). Dietary options for rodents in the study of obesity. Nutrients.

[23] Pichon L., Huneau J.-F., Fromentin G., Tomé D. (2006). A High-Protein, High-Fat, Carbohydrate-Free Diet Reduces Energy Intake, Hepatic Lipogenesis, and Adiposity in Rats. J. Nutr..

[24] Oliver P., Caimari A., Díaz-Rúa R., Palou A. (2012). Diet-induced obesity affects expression of adiponutrin/PNPLA3 and adipose triglyceride lipase, two members of the same family. Int. J. Obes..

[25] Mantzoros C. (2004). Role of leptin in reproduction. Curr. Opin. Lipidol..

[26] Paniagua J.A. (2016). Nutrition, insulin resistance and dysfunctional adipose tissue determine the different components of metabolic syndrome. World J. Diabetes.

[27] Myers M.G.J., Leibel R.L., Seeley R.J., Schwartz M.W. (2011). Obesity and Leptin Resistance: Distinguishing Cause from Effect. Trends Endocrinol. Metab..

[28] Labyb M., Chrétien C., Caillon A., Rohner-Jeanrenaud F., Altirriba J. (2019). Oxytocin administration alleviates acute but not chronic leptin resistance of diet-induced obese mice. Int. J. Mol. Sci..

[29] Farooq R., Amin S., Hayat Bhat M., Malik R., Wani H.A., Majid S. (2017). Type 2 diabetes and metabolic syndrome–adipokine levels and effect of drugs. Gynecol. Endocrinol..

[30] Al-Suhaimi E.A., Shehzad A. (2013). Leptin, resistin and visfatin: The missing link between endocrine metabolic disorders and immunity. Eur. J. Med. Res..

[31] Morris K. (2005). Resistin links obesity to type 2 diabetes. Lancet.

[32] Park H.K., Kwak M.K., Kim H.J., Ahima R.S. (2017). Linking resistin, inflammation, and cardiometabolic diseases. Korean J. Intern. Med..

[33] Xue L.N., Wang X.Y., Tan Y., Lin M., Zhang W., Xu K.Q. (2015). Significance of resistin expression in acute pancreatitis. Exp. Ther. Med..

