# Supplementary material for: The Long-Term Effects of High-Fat and High-Protein Diets on the Metabolic and Endocrine Activity of Adipocytes in Rats
Source: Biology (Basel). 2021 Apr 17;10(4):339. doi: 10.3390/biology10040339 (PMC8073757; doi:10.3390/biology10040339)
Supplement: Supplementary file 1 [file biology-10-00339-s001.zip › biology-1156336-supplementary.pdf]

Supplementary

Table S1. Content of adipokines in white adipose tissue (Pt-peritesticular; Sc-subcutaneous) and rat pancreas (Pc) on standard (SD), high protein (HPD), and high fat (HFD) diets on 60 and 120 days.

Parameter	60 d			120 d		
	SD	HPD	HFD	SD	HPD	HFD
Leptin Pt (ng/g)	35.8±4.5	29.7±3.3	55.3±16.2	54.9±8.2	36.7±7.2*a	101.3±18.1a
Leptin Sc (ng/g)	20.4±4.4	18.4±5.0	23.4±2.2	36.8±8.6	31.0±5.2a	72.0±10.1*a
Resistin Pt (ng/g)	120.7±11.3	83.3±7.0*	102.5±22.5	52.5±11.3	51.1±3.4	45.0±9.5
Resistin Sc (ng/g)	34.6±7.0	28.1±9.2	30.9±4.6	25.7±6.6	4.8±0.8*a	11.6±1.3*a
Resistin Pc (ng/g)	0.49±0.13	0.73±0.18*a	0.36±0.08a	0.17±0.04	0.45±0.13*	0.28±0.14
Visfatin Pt (ng/g)	112.4±4.4	141.5±14.3	130.5±7.3	129.9±12.7	127.4±6.5	136.0±7.4
Visfatin Sc (ng/g)	165.9±28.1	146.2±10.6	168.1±14.7	196.3±24.9	193.7±34.1	193.7±39.9

In the table are given arithmetic means ± SEM for n = 8; statistically significant differences ($p < 0.05$) between the experimental groups and the corresponding control are marked in rows by *, whereas between the experimental groups are marked by this same letter (a).

Method

Adipokines content in tissues

Tissues fragments obtained from rats were homogenized (using OMNI TISSUE HOMOGENIZER (TH)) in lysis buffer containing 20 mM Tris (pH 8.0), 137 mM NaCl, 10% glycerol, 1% Nonidet P-40, 10 mM ethylenediaminetetra acetic acid, 100 mM NaF, 1 mM phenylmethylsulfonylfluoride, 0.25 TIU/ml aprotinin, and 10 mg/ml leupeptin. Then samples were incubated on ice for 20 minutes. After this time samples were centrifuged at 14 000 x g for 15 minutes at 4˚C. The supernatant was kept in ultra-freezer (-80°C) for future analysis. The levels of resistin and leptin were determined using kits described in the main part of the manuscript. Visfatin content was determined using Visfatin EIA Kit - (cat. no. EIA-VIS-1, RayBiotech Life, USA).

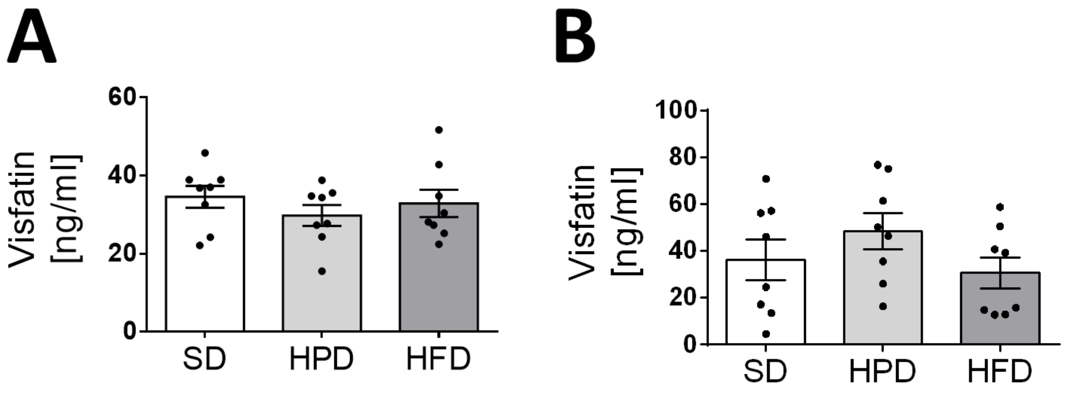

Figure S1. Effect of HPD and HFD on visfatin serum level after 60 (**A**) and 120 days (**B**) of diet treatment.
